# Supplementary material for: Higher-order interactions enhance the latitudinal tree diversity gradient
Source: Nature. 2026 Apr 29;653(8114):433–8. doi: 10.1038/s41586-026-10434-6 (PMC13171435; doi:10.1038/s41586-026-10434-6)
Supplement: Supplementary file 1 — Supplementary text, references, Tables 2–10 and Figs. 1–12. [file 41586_2026_10434_MOESM1_ESM.pdf]

nature portfolio

https://doi.org/10.1038/s41586-026-10434-6

Supplementary information

Higher-order interactions enhance the latitudinal tree diversity gradient

In the format provided by the
authors and unedited

Supplementary Information for

Higher-order interactions enhance the latitudinal tree diversity gradient

Yuanzhi Li (李远智)[1#], Junli Xiao (肖俊丽)[1#], Yuan Jiang (江园)[1], Joseph S. Wright[2], Margaret M. Mayfield[3], Oscar Godoy[4], Alfonso Alonso[5], Kristina J. Anderson-Teixeira[2,6], Jennifer Baltzer[7], Joseph D. Birch[8], Pulchérie Bissiengou[9], Norm A. Bourg[6], Warren Brockelman[10], David F.R.P. Burslem[11], Min Cao (曹敏)[12], Keith Clay[13], Stuart J. Davies[14], Qingqing Du (杜晴晴)[15], Sisira Ediriweera[16], Anna Feistner[17], Edwino Fernando[18], Gregory S. Gilbert[19], Zhanqing Hao (郝占庆)[20], Jan Holík[21], Mingxi Jiang (江明喜)[22], Guangze Jin (金光泽)[23], Daniel J. Johnson[24], Alexander S. Jones[25], Kamil Král[21], Andrew J. Larson[26], Buhang Li (李步杭)[27], Juyu Lian (练琚瑜)[28], Luxiang Lin (林露湘)[12], Feng Liu (刘峰)[29], Yu Liu (刘宇)[30], Zhili Liu (刘志理)[23], James A. Lutz[31], Keping Ma (马克平)[32], Sean McMahon[2,33], William McShea[6], Hervé Roland Memiaghe[34], Xiangcheng Mi (米湘成)[32], Jonathan A. Myers[35], Musalmah Nasardin[36], Anuttara Nathalang[10], Michael J. O'Brien[37], Nestor Engone Obiang[38], Geoffrey Parker[33], Richard P. Phillips[39], Xiujuan Qiao (乔秀娟)[22], Haibao Ren (任海保)[32], Glen Reynolds[40], Lillian JenniferV. Rodriguez[41], Pavel Šamonil[21], Guochun Shen (沈国春)[42], Zufei Shu (束祖飞)[43], Jessica Shue[2,33], Mark E. Swanson[44], Jill Thompson[45], María Uriarte[46], Xihua Wang (王希华)[42], Xugao Wang (王绪高)[47], Youshi Wang (王酉石)[27], Tze Leong Yao[36], Wanhui Ye (叶万辉)[28], Mingjian Yu (于明坚)[48], Minhua Zhang (张敏华)[30], Yan Zhu (祝燕)[15], Jess Zimmerman[49], Fangliang He (何芳良)[30,50], Chengjin Chu (储诚进)[1*]

Contents

Supplementary Text 2
Supplementary References.. 12
Supplementary Tables 14
Supplementary Figures 23

Supplementary Text

1. Calculating pairwise and higher-order neighbor crowding indices

Following our previous work on estimating higher-order interactions in a temperate forest[1], here we summarize the methods of computing pairwise and higher-order neighbor crowding indices. The growth and survival of a focal tree i_f (tree f of species i) depend not only on its neighbors (e.g., j_p in Fig. S1) but also on its neighbor's neighbors (e.g., k_q in Fig. S1). The total pairwise neighbor crowding index of focal tree i_f, denoted as n_{t,i_f}, counts all neighbors (j_p) within distance R, weighted by their sizes (measured by diameter at breast height, DBH_{j_p}) and distances to focal tree ($d[i_f, j_p]$):

$$i_f \leftarrow j_p: n_{t,i_f} = \sum_{p=1}^{N_{i_f}} \frac{DBH_{j_p}^{u}}{d[i_f, j_p]^{v}} \quad \text{(S1)}$$

N_{i_f} is the total number of neighbors within distance R of a focal tree, i_f. Parameter u and v are the size- and distance-scaling exponents, implying that distant and small neighbors exert weak effects[2,3]. When $u = 0$ and $v = 0$, n_{tot,i_f} simply equals N_{i_f}. The total pairwise neighbor crowding index can be further decomposed into conspecific (n_{i,i_f}) and heterospecific (n_{h,i_f}) components:

$$n_{t,i_f} = n_{i,i_f} + n_{h,i_f} \quad \text{(S2)}$$

The index n_{i,i_f} counts all conspecific neighbors ($j = i$):

$$n_{i,i_f} = \sum_{p=1}^{N_{i,i_f}} \frac{DBH_{i_p}^{u}}{d[i_f, i_p]^{v}} \quad \text{(S3)}$$

The n_{h,i_f} counts all heterospecific neighbors ($j = h$):

$$n_{h,i_f} = \sum_{p=1}^{N_{h,i_f}} \frac{DBH_{h_p}^{u}}{d[i_f, h_p]^{v}} \quad \text{(S4)}$$

The total higher-order neighbor crowding index of focal tree i_f, denoted as n_{tt,i_f}, counts all combinations of its neighbors (j_p) and those neighbors' neighbors (k_q), weighted by their sizes (DBH_{j_p}, DBH_{k_q}) and distances ($d[i_f, j_p]$, $d[j_p, k_q]$):

$$n_{tt,i_f} = \sum_{p=1}^{N_{i_f}} \left(\frac{DBH_{j_p}^u}{d[i_f, j_p]^v} \cdot \sum_{q=1}^{N_{j_p}} \frac{DBH_{k_q}^u}{d[j_p, k_q]^v} \right) = \sum_{p=1}^{N_{i_f}} \left(\frac{DBH_{i_p}^u}{d[i_f, j_p]^v} \cdot n_{t,j_p} \right) \quad \text{(S5)}$$

The total higher-order neighbor crowding index can be further decomposed into four components:

$$n_{tt,i_f} = n_{ii,i_f} + n_{ih,i_f} + n_{hi,i_f} + n_{hh,i_f} \quad \text{(S6)}$$

(i) n_{ii,i_f} is higher-order neighbor crowding index when $j = i$ and $k = i$ (i.e., intraspecific pairwise interactions modified by conspecific neighbors):

$$n_{ii,i_f} = \sum_{p=1}^{N_{i,i_f}} \left(\frac{DBH_{i_p}^u}{d[i_f, i_p]^v} \cdot \sum_{q=1}^{N_{i,i_p}} \frac{DBH_{i_q}^u}{d[i_p, i_q]^v} \right) = \sum_{p=1}^{N_{i,i_f}} \left(\frac{DBH_{i_p}^u}{d[i_f, i_p]^v} \cdot n_{i,i_p} \right) \quad \text{(S7)}$$

(ii) n_{ih,i_f} is higher-order neighbor crowding index when $j = i$ and $k \neq i$ (i.e., intraspecific pairwise interactions modified by heterospecific neighbors):

$$n_{ih,i_f} = \sum_{p=1}^{N_{i,i_f}} \left(\frac{DBH_{i_p}^u}{d[i_f, i_p]^v} \cdot \sum_{q=1}^{N_{h,i_p}} \frac{DBH_{h_q}^u}{d[i_p, h_q]^v} \right) = \sum_{p=1}^{N_{i,i_f}} \left(\frac{DBH_{i_p}^u}{d[i_f, i_p]^v} \cdot n_{h,i_p} \right) \quad \text{(S8)}$$

(iii) n_{hi,i_f} is higher-order neighbor crowding index when $j \neq i$ and $k = i$ (i.e., interspecific pairwise interactions modified by conspecific neighbors):

$$n_{hi,i_f} = \sum_{p=1}^{N_{h,i_f}} \left(\frac{DBH_{h_p}^u}{d[i_f, h_p]^v} \cdot \sum_{q=1}^{N_{i,h_p}} \frac{DBH_{i_q}^u}{d[h_p, i_q]^v} \right) = \sum_{p=1}^{N_{h,i_f}} \left(\frac{DBH_{h_p}^u}{d[i_f, h_p]^v} \cdot n_{i,h_p} \right) \quad \text{(S9)}$$

(iv) n_{hh,i_f} is higher-order neighbor crowding index when $j \neq i$ and $k \neq i$ (i.e., interspecific pairwise interactions modified by heterospecific neighbors):

$$n_{hh,i_f} = \sum_{p=1}^{N_{h,i_f}} \left(\frac{DBH_{h_p}^u}{d[i_f, h_p]^v} \cdot \sum_{q=1}^{N_{h,h_p}} \frac{DBH_{h_q}^u}{d[h_p, h_q]^v} \right) = \sum_{p=1}^{N_{h,i_f}} \left(\frac{DBH_{h_p}^u}{d[i_f, h_p]^v} \cdot n_{h,h_p} \right) \quad \text{(S10)}$$

The empirical correlations between the pairwise crowding indices (n_{i,i_f} and n_{h,i_f}) and higher-order indices (n_{ii,i_f}, n_{ih,i_f}, n_{hi,i_f} and n_{hh,i_f}) are shown in Fig. S2. Strong correlations between n_{i,i_f} and n_{ii,i_f}, and between n_{h,i_f} and n_{hh,i_f} arise because n_{ii,i_f} and n_{hh,i_f} are approximately the square of n_{i,i_f} and n_{h,i_f}, respectively.

2. Robustness of results for growth models

2.1 Results are robust to different parameter settings

To test the robustness of our findings, we considered type II functional response of HOIs, in which their effects saturate with increasing neighborhood crowding[4]:

$$HOI_{i_f} = \frac{\beta_{iii} \cdot n_{ii,i_f}}{1 + m \cdot n_{ii,i_f}} + \frac{\beta_{iih} \cdot n_{ih,i_f}}{1 + m \cdot n_{ih,i_f}} + \frac{\beta_{ihi} \cdot n_{hi,i_f}}{1 + m \cdot n_{hi,i_f}} + \frac{\beta_{ihh} \cdot n_{hh,i_f}}{1 + m \cdot n_{hh,i_f}} \quad \text{(S11)}$$

The higher-order effects saturate at a rate controlled by the parameter m. Equation (4) in the main text is a special case of equation (S11) when $m = 0$.

To make the coefficients of pairwise and higher-order interactions comparable among different species in different plots, we set radius (R), scaling parameter (u and v), and saturating parameter (m) to be the same for all species rather than find species-specific optimum values. To test the generality of our analyses, we tried several radii (e.g., R = 10 m, 20 m) and scaling parameters (u = 0, 1, 2 and v = 0, 1, 2) as commonly practiced in forest ecology studies[5-7]. For the saturating parameter (m), we first conducted nonlinear regression (S11) for the growth model to estimate the species-specific saturating parameter (m). The estimated species-specific m ranged from 0.9×10^{-5} to 2×10^{-3} which served as the range to set m values. In the test, we sequentially sampled five values within this range and assigned them to all species. We also included the special case $m = 0$.

The results were generally consistent across different parameter settings (Supplementary Tables 2-5). They show HOIs are prevalent and decline with latitude regardless of the parameter settings. Therefore, in the main text we only reported results with the parameter setting of R = 20 m, u = 1, v = 1 and m = 0, which had the lowest AIC value compared to other parameter settings.

2.2 Results are robust to spatial autocorrelation

We used the R package DHARMa[8] to test for spatial autocorrelation. Spatial autocorrelation was significantly detected for 27% of the growth models. To account for this spatial autocorrelation, we divided each plot into quadrats of 20 m × 20 m and included quadrats as a random intercept. Warning messages of singular fit were detected in 12% of the growth models. For the models without the warning massage, we found that the interaction coefficients (α and β) estimated from models with and without considering spatial autocorrelation were very similar

(Fig. S3). We therefore reported results based on models without considering spatial autocorrelation, as more species could be included without changing overall conclusions.

2.3 Results are robust to uncertainty of interaction coefficients

The coefficients of pairwise and higher-order interactions were estimated from growth models with inherent uncertainty (estimate error). We evaluated the sensitivity of our results to this uncertainty using parametric bootstrapping. Specifically, each interaction coefficient was randomly sampled 1000 times from a normal distribution, with the mean equal to its estimated value and the standard deviation equal to its standard error from the growth models. We then repeated interaction-latitude regressions 1000 times and calculated the percentage of relationships that showed the strength of the interactions significantly declined with latitude. The latitudinal changes in pairwise and higher-order interactions were robust to estimated uncertainty (Supplementary Table 6).

We further tested effects of the uncertainty on relationship between relative change in growth rate with species abundance and latitude. Specifically, we generated 1000 sets of coefficients from a multivariate normal distribution based on the unconditional variance-covariance matrix of the fitted growth model. For each set, we calculated the relative change in growth rate caused by cumulative effects of pairwise interactions and HOIs, and then related them to species abundance and latitudes. The relationships between relative change in growth rate with species abundance and latitude were also robust to the uncertainty of the coefficients (Supplementary Table 7).

2.4 Results are robust to size of trees

In our study, the small trees ($DBH < 10$ cm) dominate our 1543 species–plot combinations, with an average proportion of 77% (ranging from 46% to 97% across the 32 forest plots). To explore the influence of tree size (DBH) on pairwise and higher-order interactions, as well as their latitudinal trends, we fitted the growth models for small trees ($DBH < 10$ cm) and large trees ($DBH \geq 10$ cm), separately. We found that HOIs are prevalent for both large and small trees (Fig. S4). The pairwise and higher-order interactions (except β_{ihh}) decline with latitude for both small trees (Fig. S5) and large trees (Fig. S6). In addition, we calculated the relative changes in growth rate caused by cumulative effects of pairwise interactions and HOIs for both

large and small trees, and fitted their relationship with species abundance and latitude. For both size classes, the relative changes in growth rate caused by cumulative effects of pairwise interactions and HOIs both shift from beneficial for rare species to detrimental for common species (Figs. S7 and S8). Moreover, the stabilizing effect of pairwise interactions remains relatively constant across latitudes for both small and large trees (Supplementary Tables 8 and 9). In contrast, the stabilizing effect of HOIs becomes weaker towards higher latitudes for small trees (Supplementary Table 8) but changes little for large trees (Supplementary Table 9). These results are consistent with those for all trees combined, and thus we only show combined effects in the main text (Table 1, Figs. 4 and 5 in main text).

3. Upscaling interaction coefficients and structural stability analyses

To further explore the contributions of pairwise interactions and HOIs to the latitudinal diversity gradient, we adopt a recently developed structural approach for understanding multispecies coexistence[9,10]. This approach has several methodological advantages. First, it integrates complex biotic interactions into a metric of "structural stability" that measures the maximum environmental perturbations that a community can withstand without losing species (i.e., the probability that a community/species can persist). Second, it allows us to account for different types of biotic interactions (competitive or facilitative, pairwise or HOIs), simultaneously. Third, it enables us to quantify the relative contribution of pairwise interactions and HOIs by comparing the difference in structural stability estimated from $\alpha_{ij,true}$ and $\alpha_{ij,modified}$.

The individual-level interaction coefficients (α_{ii}, α_{ih}, β_{iii}, β_{iih}, β_{ihi} and β_{ihh}) that are estimated directly from the demographic models (equation 1 and 2) at neighborhood scale need to be upscaled to population-level interaction coefficients (α'_{ii} , α'_{ih}, β'_{iii}, β'_{iih}, β'_{ihi} and β'_{ihh}) before being applied to this structural stability analysis. Although methods have recently been developed to upscale pairwise interaction coefficients[11,12], upscaling higher-order interactions remains a challenge. Here, we propose an approximation.

Following Wiegand et al. (2025)[11], we illustrate the process using a simple population model with three assumptions: (1) all individuals could reproduce (not considering growth of trees), (2) reproduction is density-independent with a constant rate for each species (r_i), (3) survival rate (surv_i) is density-dependent. The population growth of a species is determined by the difference between reproduction and mortality ($1 - \mathrm{surv}_i$):

$$\frac{dN_i}{dt} = N_i(r_i - (1 - \mathrm{surv}_i)) \quad \text{(S12)}$$

The survival rate can be calculated as the average of individual survival probability:

$$\mathrm{surv}_i = \sum_{f=1}^{N_i} \mathrm{surv}_{i_f} / N_i = \overline{\mathrm{surv}}_{i_f} \quad \text{(S13)}$$

where N_i is the abundance of species i, and surv_{i_f} is the survival probability of tree f of species i that is determine by equation (2) in the main text. For convenience, we rewrite the survival model as:

$$\mathrm{surv}_{i_f} = \frac{1}{1 + ae^{x}} \quad \text{(S14)}$$

where $a = e^{\lambda_i + \gamma_1 \cdot DBH_{i_f}^{-1} + \gamma_2 \cdot DBH_{i_f} + \gamma_3 \cdot DBH_{i_f}^{2}}$ and $x = PAIR_{i_f} + HOI_{i_f}$ are density-independent and density-dependent effects on survival probability, respectively. Equation (S14) could be approximated by its linearization form:

$$\mathrm{surv}_{i_f} \approx \frac{1}{1 + a} - \frac{a}{(1 + a)^2} x \quad \text{(S15)}$$

Given that the correlation a and x is low for most species (Fig. S9), their effects on survival probability are assumed to be independent for simplification. Then the average survival probability could be calculated as:

$$\overline{\mathrm{surv}}_{i_f} = \overline{\frac{1}{1 + a}} - \overline{\frac{a}{(1 + a)^2}} \bar{x} \quad \text{(S16)}$$

Combining equation (3) and (4) in the main text, we have:

$$\bar{x} = \alpha_{ii}\bar{n}_{ii} + \alpha_{ih}\bar{n}_{ih} + \beta_{iii}\bar{n}_{iii} + \beta_{iih}\bar{n}_{iih} + \beta_{ihi}\bar{n}_{ihi} + \beta_{ihh}\bar{n}_{ihh} \quad \text{(S16)}$$

where $\bar{n}_{ii} = \bar{n}_{i,i_f}$, $\bar{n}_{ih} = \bar{n}_{h,i_f}$, $\bar{n}_{iii} = \bar{n}_{ii,i_f}$, $\bar{n}_{iih} = \bar{n}_{ih,i_f}$, $\bar{n}_{ihi} = \bar{n}_{hi,i_f}$ and $\bar{n}_{ihh} = \bar{n}_{hh,i_f}$ are the average pairwise and high-order neighborhood crowding indices (Supplementary Note 1). We also calculated the expected pairwise and high-order neighborhood crowding indices under complete spatial randomness (CSR). The expected pairwise conspecific and heterospecific neighborhood crowding indices under CSR (e_{ii} and e_{ih}) are calculated as[11,12]:

$$\begin{cases} e_{ii} = E\left(n_{i,i_f}\right) = cN_i \\ e_{ih} = E\left(n_{h,i_f}\right) = cN_h \end{cases} \quad \text{(S17)}$$

where N_h is the abundance of all species other than species i; c is a constant depending on choice of size and distance scaling parameter u and v (Supplementary Table 10). Under CSR, the neighbor crowding indices of focal tree i_f (n_{i,i_f} and n_{h,i_f}) are independent of the neighbor crowding indices of its neighbor tree i_p (n_{i,i_p} and n_{h,i_p}) or h_p (n_{i,h_p} and n_{h,h_p}). Therefore, the expected higher-order neighborhood crowding indices under CSR (e_{iii}, e_{iih}, e_{ihi} and e_{ihh}), following equations S7-S10, can be calculated as:

$$
\begin{cases}
e_{iii} = E\left(\sum_{p=1}^{N_{i,i_f}} \frac{DBH_{i_p}^{u}}{d\left[i_f, i_p\right]^{v}} \cdot n_{i,i_p}\right) = E\left(n_{i,i_f}\right) E\left(n_{i,i_p}\right) = c^2 N_i^2 \\
e_{iih} = E\left(\sum_{p=1}^{N_{i,i_f}} \frac{DBH_{i_p}^{u}}{d\left[i_f, i_p\right]^{v}} \cdot n_{h,i_p}\right) = E\left(n_{i,i_f}\right) E\left(n_{h,i_{\square\square}}\right) = c^2 N_i N_h \\
e_{ihi} = E\left(\sum_{p=1}^{N_{i,i_f}} \frac{DBH_{h_p}^{u}}{d\left[i_f, h_p\right]^{v}} \cdot n_{i,h_p}\right) = E\left(n_{h,i_f}\right) E\left(n_{i,h_p}\right) = c^2 N_i N_h \\
e_{ihh} = E\left(\sum_{p=1}^{N_{h,i_f}} \frac{DBH_{h_p}^{u}}{d\left[i_f, h_p\right]^{v}} \cdot n_{h,h_p}\right) = E\left(n_{h,i_f}\right) E\left(n_{h,h_p}\right) = c^2 N_h^2
\end{cases}
\quad (S18)
$$

We next calculate the aggregation indices (k_{ii}, k_{ih}, k_{iii}, k_{iih}, k_{ihi} and k_{ihh}) as the ratio of observed neighborhood crowding indices to the expected neighborhood crowding indices under CSR:

$$
\begin{cases}
k_{ii} = \frac{\bar{n}_{ii}}{e_{ii}} \\
k_{ih} = \frac{\bar{n}_{ih}}{e_{ih}} \\
k_{iii} = \frac{\bar{n}_{iii}}{e_{iii}} \\
k_{iih} = \frac{\bar{n}_{iih}}{e_{iih}} \\
k_{ihi} = \frac{\bar{n}_{ihi}}{e_{ihi}} \\
k_{ihh} = \frac{\bar{n}_{ihh}}{e_{ihh}}
\end{cases}
\quad (S19)
$$

Substituting the equation (S17-19) to equation (16), we have:

$$\bar{x} = ck_{ii}\alpha_{ii}N_i + ck_{ih}\alpha_{ih}N_h + c^2 k_{iii}\beta_{iii}N_i^2 + c^2 k_{iih}\beta_{iih}N_i N_h + c^2 k_{ihi}\beta_{ihi}N_i N_h + c^2 k_{iii}\beta_{iii}N_h^2 \quad (S20)$$

We finally obtain an approximate solution for upscaling the individual-level interaction coefficients to population-level interaction coefficients:

$$\begin{cases} \alpha'_{ii} = ck_{ii}\alpha_{ii} \\ \alpha'_{ih} = ck_{ih}\alpha_{ih} \\ \beta'_{iii} = c^2 k_{iii}\beta_{iii} \\ \beta'_{iih} = c^2 k_{iih}\beta_{iih} \\ \beta'_{ihi} = c^2 k_{ihi}\beta_{ihi} \\ \beta'_{ihh} = c^2 k_{ihh}\beta_{ihh} \end{cases} \quad (S21)$$

and then we obtain the generalized Lotka-Volterra population dynamical model with inclusion of HOIs:

$$\frac{dN_i}{dt} = N_i\left(r_i - 1 + \overline{\frac{1}{1+a}} - \overline{\frac{a}{(1+a)^2}}\left(N_i + \alpha'_{ih}N_h + \beta'_{iii}N_i^2 + \beta'_{iih}N_iN_h + \beta'_{ihi}N_hN_i + \beta'_{ihh}N_h^2\right)\right) \quad (S22)$$

Although structural stability is a promising way of assessing the contribution of biotic interactions to the latitudinal diversity gradient with the advantages stated above, no method is yet available to evaluate structural stability with the inclusion of HOIs (e.g. model S22)[13]. Instead, structural stability based on $\alpha_{modified}$ that implicitly incorporates HOIs can provide an approximate solution[14]. The structural stability of a community with a given interaction matrix of n species ($A_{n\times n}$) is calculated by the cumulative function of a multivariate Gaussian distribution[9,10]:

$$\Omega(A) = \frac{1}{(2\pi)^{\frac{n}{2}}\sqrt{\det(A)}}\int_{R^n \geq 0} e^{-\frac{1}{2}\boldsymbol{x}^T A^T A \boldsymbol{x}} d\boldsymbol{x}\,;\, \omega(A) = \sqrt[n]{\Omega(A)} \quad (S23)$$

$\Omega(A)$ measures the probability of the persistence of a given community (i.e., all species maintain a positive equilibrium). $\omega(A)$ translates the probabilistic interpretation of feasibility from the community to the species level, and implies that a randomly chosen species from the given community is feasible. Given that structural stability ($\omega(A_{n\times n})$) declines intrinsically with species richness (Fig. S10), we constructed null models to make it comparable across plots with different species richness following the steps below (Fig. S11). (1) We calculated the mean (μ) and standard deviation (σ) of intraspecific (μ_{ii} and σ_{ii} of α_{ii}) and interspecific pairwise interactions (μ_{ih} and σ_{ih} of α_{ih}) over species across all forest plots. (2) We constructed 100

random interaction matrices for each plot with the same number of tree species $A = [\alpha_{ij}]_{n\times n}$, where α_{ii} and α_{ih} are randomly sampled from normal distributions with the mean and standard deviation calculated over all plots $\left(\alpha_{ii} \sim N(\mu_{ii}, \sigma_{ii}^2), \alpha_{ih} \sim N\left(\mu_{ij}, \sigma_{ih}^2\right)\right)$. (3) The mean and standard deviation of the structural stability over the 100 null matrices ($\mu(\omega_{null})$ and $\sigma(\omega_{null})$) were calculated for each plot. (4) We also calculated the mean and standard deviation of intraspecific and interspecific pairwise interactions over species for each plot ($\mu_{ii,k}$ and $\sigma_{ii,k}$ of $\alpha_{ii,k}$, $\mu_{ih,k}$ and $\sigma_{ih,k}$ of $\alpha_{ih,k}$ for plot *k*). The number of tree species involved varies greatly across the plots (2 to 257 species) and the standard deviation is underestimated for plots with fewer species, we thus adjusted the standard deviation of pairwise interactions in plot *k* with n_k species:

$$\sigma_{k.adj} = \sqrt{\frac{n_k - 1}{2}} \frac{\Gamma\left(\frac{n_k - 1}{2}\right)}{\Gamma\left(\frac{n_k}{2}\right)} \sigma_k \quad (S24)$$

where Γ is the gamma function. (5) We generated 100 interaction matrices for each plot with the same number of tree species $A = [\alpha_{ij}]_{n\times n}$, where α_{ii} and α_{ih} are randomly sampled from a normal distribution with the mean and standard deviation calculated for each plot $\left(\alpha_{ii} \sim N\left(\mu_{ii,k}, \sigma_{ii,k}^2\right), \alpha_{ih} \sim N\left(\mu_{ih,k}, \sigma_{ih,k}^2\right)\right)$. (6) The mean and standard deviation of the structural stability over the 100 matrices generated from the observed pairwise interaction summary (mean and standard deviation) in each plot ($\mu(\omega_{ob})$ and $\sigma(\omega_{ob})$) were calculated. (7) The standard effect size (SES) of ω for each plot was calculated:

$$SES_\omega = \frac{\mu(\omega_{ob}) - \mu(\omega_{null})}{\sigma(\omega_{null})} \quad (S25)$$

The above steps were repeated for pairwise interactions $\alpha_{ij,true}$ and $\alpha_{ij,modified}$. Finally, we tested Spearman correlations between the standard effect size of structural stability and species richness.

The results are presented in Fig. S12. The standard effect size of structural stability based on α_{true} and $\alpha_{modified}$ is marginally positively correlated with species richness.

Supplementary References

1 Li, Y. *et al.* Beyond direct neighbourhood effects: higher-order interactions improve modelling and predicting tree survival and growth. *National Science Review* **8**, nwaa244 (2021).

2 Uriarte, M., Canham, C. D., Thompson, J. & Zimmerman, J. K. A neighborhood analysis of tree growth and survival in a hurricane-driven tropical forest. *Ecological monographs* **74**, 591-614 (2004).

3 Canham, C. D., LePage, P. T. & Coates, K. D. A neighborhood analysis of canopy tree competition: effects of shading versus crowding. *Canadian Journal of Forest Research* **34**, 778-787 (2004).

4 Gibbs, T., Levin, S. A. & Levine, J. M. Coexistence in diverse communities with higher-order interactions. *Proceedings of the National Academy of Sciences* **119**, e2205063119 (2022).

5 Chen, Y. *et al.* Positive effects of neighborhood complementarity on tree growth in a Neotropical forest. *Ecology* **97**, 776-785 (2016).

6 Zambrano, J. *et al.* Tree crown overlap improves predictions of the functional neighbourhood effects on tree survival and growth. *Journal of Ecology* **107**, 887-900 (2018).

7 Maes, S. L. *et al.* Environmental drivers interactively affect individual tree growth across temperate European forests. *Globle Change Biology* **25**, 201-217 (2019).

8 DHARMa: residual diagnostics for hierarchical (multi-level / mixed) regression models v. R package version 0.4.7 (2024).

9 Serguei, S. *et al.* A structural approach for understanding multispecies coexistence. *Ecological monographs* **87**, 470-486 (2017).

10 Song, C., Rohr, R. P. & Saavedra, S. A guideline to study the feasibility domain of multi-trophic and changing ecological communities. *Journal of Theoretical Biology* **450**, 30-36 (2018).

11 Wiegand, T. *et al.* Consequences of spatial patterns for coexistence in species-rich plant communities. *Nature Ecology & Evolution* **5**, 965-973 (2021).

12 Wiegand, T. *et al.* Latitudinal scaling of aggregation with abundance and coexistence in forests. *Nature* **640**, 967-973 (2025).

13 AlAdwani, M. & Saavedra, S. Feasibility conditions of ecological models: Unfolding links between model parameters. *Ecological Modelling* **466**, 109900 (2022).

14 Buche, L., Bartomeus, I. & Godoy, O. Multitrophic higher-order interactions modulate species persistence. *The American Naturalist* **203**, 458-472 (2024).

Supplementary Tables

Supplementary Table 1 | Summary information of the 32 forest plots. (Excel file: Supplementary Table 1.xlsx)

Supplementary Table 2 | Evidence of higher-order interactions in different settings of radius (*R*) and scaling parameter (*u* and *v*). The percentages of 1,543 species–plot combinations across the 32 plots for which each of the three classes of growth models are best supported (AIC at least 2 units lower than that of the two alternative models) in different settings of radius (*R*) and scaling parameter (*u* and *v*). The saturating parameter is set the same as in the main text ($m = 0$). The three types of models include NULL models (models without any biotic interactions), PAIR-only models (models includes only pairwise interactions) and the HOI-inclusive models (models include both pairwise interactions and HOIs).

R	u	v	NULL	PAIR-only model	HOI-inclusive model
10	0	0	49%	26%	24%
10	1	1	39%	34%	27%
10	2	2	73%	12%	14%
20	0	0	43%	19%	37%
20	2	1	45%	20%	35%
20	2	2	64%	11%	25%

Supplementary Table 3 | Evidence of higher-order interactions in different settings of saturating parameter (*m*). The percentages of 1,543 species–plot combinations across the 32 plots for which each of the three classes of growth models are best supported (AIC at least 2 units lower than that of the two alternative models) in different settings of the saturating parameter (m). The radius (R) and scaling parameter (u and v) are set the same as in the main text (R = 20 m, u = 1 and v = 1). The three types of models include NULL models (models without including biotic interactions), PAIR-only models (models includes only pairwise interactions) and the HOI-inclusive model models (models include both pairwise interactions and HOIs).

m	NULL	PAIR-only model	HOI-inclusive model
8.93E-05	33%	27%	39%
2.03E-04	33%	27%	40%
4.05E-04	33%	27%	39%
9.95E-04	34%	27%	39%
2.11E-03	34%	28%	37%

Supplementary Table 4 | Latitudinal changes in pairwise and higher-order interactions in different settings of radius (*R*) and scaling parameter (*u* and *v*). The saturating parameter *m* is set the same as in the main text (m = 0). The species-level pairwise and higher-order interactions are related to absolute latitude separately for competition ($-\alpha, -\beta$) and facilitation ($+\alpha, +\beta$) using exponential regressions ($y = a \cdot e^{bx}$). Significance of the regression coefficients was assessed using two-sided *t*-test. Symbols "$-$" and "$+$" represent a significant decline and increase in the strength of interactions with increasing latitudes, respectively, while "0" indicates non-significant latitudinal change.

Model	Parameter	***R*=10 *u*=0 *v*=0**	***R*=10 *u*=1 *v*=1**	***R*=10 *u*=2 *v*=2**	***R*=20 *u*=0 *v*=0**	***R*=10 *u*=2 *v*=1**	***R*=20 *u*=2 *v*=2**
PAIR-only model	$+\alpha_{ii}$	-	-	-	-	0	-
PAIR-only model	$-\alpha_{ii}$	-	-	-	-	-	-
PAIR-only model	$+\alpha_{ih}$	+	0	-	+	+	0
PAIR-only model	$-\alpha_{ih}$	0	-	0	0	+	0
HOI-inclusive model	$+\alpha_{ii}$	-	-	-	-	-	-
HOI-inclusive model	$-\alpha_{ii}$	-	-	-	-	-	-
HOI-inclusive model	$+\alpha_{ih}$	0	-	0	0	0	0
HOI-inclusive model	$-\alpha_{ih}$	0	-	+	0	+	0
HOI-inclusive model	$+\beta_{iii}$	-	-	-	-	-	-
HOI-inclusive model	$-\beta_{iii}$	-	-	-	-	-	-
HOI-inclusive model	$+\beta_{iih}$	-	-	-	-	-	-
HOI-inclusive model	$-\beta_{iih}$	-	-	-	-	-	-
HOI-inclusive model	$+\beta_{ihi}$	0	-	-	0	-	-
HOI-inclusive model	$-\beta_{ihi}$	0	-	-	0	-	-
HOI-inclusive model	$+\beta_{ihh}$	+	0	0	+	+	0
HOI-inclusive model	$-\beta_{ihh}$	+	-	0	+	0	0

Supplementary Table 5 | Latitudinal changes in pairwise and higher-order interactions in different settings of saturating parameter (*m*). The radius (*R*) and scaling parameter (*u* and *v*) are set the same as in the main text (*R* = 20 m, *u* = 1 and *v* = 1). The species-level pairwise interactions and HOIs are related to absolute latitude separately for competition ($-\alpha, -\beta$) and facilitation ($+\alpha, +\beta$) using weighted least squares exponential regressions. Significance of the regression coefficients was assessed using two-sided *t*-test. Symbols "$-$" and "$+$" represent a significant decline and increase in strength of interactions with increasing latitudes respectively, while "0" indicates non-significant latitudinal change.

**Model**	**Parameter**	***m***				
		8.93E-05	2.03E-04	4.05E-04	9.95E-04	2.11E-03
PAIR-only model	$+\alpha_{ii}$	-	-	-	-	-
PAIR-only model	$-\alpha_{ii}$	-	-	-	-	-
PAIR-only model	$+\alpha_{ih}$	0	0	0	0	0
PAIR-only model	$-\alpha_{ih}$	-	-	-	-	-
HOI-inclusive model	$+\alpha_{ii}$	-	-	-	-	-
HOI-inclusive model	$-\alpha_{ii}$	-	-	-	-	-
HOI-inclusive model	$+\alpha_{ih}$	-	-	-	-	-
HOI-inclusive model	$-\alpha_{ih}$	-	-	-	-	-
HOI-inclusive model	$+\beta_{iii}$	-	-	-	-	-
HOI-inclusive model	$-\beta_{iii}$	-	-	-	-	-
HOI-inclusive model	$+\beta_{iih}$	-	-	-	0	+
HOI-inclusive model	$-\beta_{iih}$	-	-	-	0	+
HOI-inclusive model	$+\beta_{ihi}$	-	-	-	0	+
HOI-inclusive model	$-\beta_{ihi}$	-	-	0	+	+
HOI-inclusive model	$+\beta_{ihh}$	-	-	-	-	-
HOI-inclusive model	$-\beta_{ihh}$	-	-	-	-	-

Supplementary Table 6 | Latitudinal changes in pairwise and higher-order interactions accounting for the uncertainty of the estimation of coefficients. The percentage of relationships for which the strength of the interactions significantly declines, increases, or does not change with absolute latitude in 1000 random samples from normal distribution. Regressions for each type of interactions are conducted separately for competition (-) and facilitation (+) using exponential regressions. Significance of the regression coefficients was assessed using two-sided *t*-test. The radius (*R*), scaling parameter (*u* and *v*) and saturating parameter (m) are set the same as in the main text (*R* = 20 m, *u* = 1, *v* = 1 and *m* = 0).

**Model**	**Parameter**	**Decline**	**Increase**	**No significant change**
PAIR-only model	$+\alpha_{ii}$	100%	0%	0%
PAIR-only model	$-\alpha_{ii}$	100%	0%	0%
PAIR-only model	$+\alpha_{ih}$	11%	11%	78%
PAIR-only model	$-\alpha_{ih}$	50%	0%	50%
HOI-inclusive model	$+\alpha_{ii}$	100%	0%	0%
HOI-inclusive model	$-\alpha_{ii}$	100%	0%	0%
HOI-inclusive model	$+\alpha_{ih}$	59%	1%	40%
HOI-inclusive model	$-\alpha_{ih}$	89%	0%	11%
HOI-inclusive model	$+\beta_{iii}$	100%	0%	0%
HOI-inclusive model	$-\beta_{iii}$	100%	0%	0%
HOI-inclusive model	$+\beta_{iih}$	100%	0%	0%
HOI-inclusive model	$-\beta_{iih}$	100%	0%	0%
HOI-inclusive model	$+\beta_{ihi}$	100%	0%	0%
HOI-inclusive model	$-\beta_{ihi}$	100%	0%	0%
HOI-inclusive model	$+\beta_{ihh}$	0%	100%	0%
HOI-inclusive model	$-\beta_{ihh}$	0%	39%	61%

Supplementary Table 7 | Latitudinal changes in cumulative effects of pairwise and higher-order interactions (HOIs) accounting for the uncertainty of the estimation of coefficients

The percentage of negative or positive relationships between relative changes in growth rate with species abundance and absolute latitude in 1000 random simulations. RC_{PAIR} and RC_{HOI} refer to relative changes in growth rate caused by cumulative effects of pairwise interactions and HOIs, respectively. The relative change in growth rate is fitted by linear models using log-transformed species abundance, absolute latitude and their interaction as predictors (N = 1,543 species). The radius (R), scaling parameter (u and v) and saturating parameter (m) are set the same as in the main text (R = 20 m, u = 1, v = 1 and m = 0).

**Response**	**Effect**	**Negative**	**Positive**
RC_{PAIR}	Intercept	2%	98%
	Abundance	95%	5%
	Latitude	80%	20%
	Abundance:Latitude	24%	76%
RC_{HOI}	Intercept	0%	100%
	Abundance	100%	0%
	Latitude	64%	36%
	Abundance:Latitude	23%	77%

Supplementary Table 8 | Summary of how relative changes in growth rate vary with species abundance and absolute latitude for small trees ($DBH < 10$ cm). RC_{PAIR} and RC_{HOI} represent relative changes in growth rate caused by cumulative effects of pairwise and higher-order interactions, respectively. The relative change in growth rate is fitted by linear models using log-transformed species abundance, absolute latitude and their interaction as predictors (*N* = 1,362 species).

Response	Effect	Estimate	Standard error	t value	*P* value
RC_{PAIR}	Intercept	1.628	0.581	2.801	0.005
	Abundance	-0.522	0.185	-2.823	0.005
	Latitude	0.005	0.030	0.158	0.875
	Abundance:Latitude	0.002	0.008	0.200	0.842
RC_{HOI}	Intercept	3.790	0.549	6.899	8.00×10^{-12}
	Abundance	-0.908	0.175	-5.199	2.31×10^{-7}
	Latitude	-0.063	0.028	-2.222	0.026
	Abundance:Latitude	0.018	0.008	2.362	0.018

Supplementary Table 9 | Summary of how relative changes in growth rate vary with species abundance and absolute latitude for large trees ($DBH \geq 10$ cm). RC_{PAIR} and RC_{HOI} represent relative changes in growth rate caused by cumulative effects of pairwise and higher-order interactions, respectively. The relative change in growth rate is fitted by linear models using log-transformed species abundance, absolute latitude and their interaction as predictors (*N* = 432 species).

Response	Effect	Estimate	Standard error	t value	*P* value
RC_{PAIR}	Intercept	2.825	0.944	2.993	0.003
	Abundance	-0.931	0.373	-2.493	0.013
	Latitude	-0.050	0.039	-1.287	0.199
	Abundance:Latitude	0.015	0.013	1.164	0.245
RC_{HOI}	Intercept	1.057	0.517	2.043	0.042
	Abundance	-0.199	0.205	-0.970	0.332
	Latitude	-0.009	0.021	-0.404	0.686
	Abundance:Latitude	0.003	0.007	0.434	0.665

Supplementary Table 10 | The constant c used in upscaling the interaction coefficients. The constant c used in upscaling of the interaction coefficients depends on the setting of parameters including the radius (R) and size- and distance scaling parameters (u and v).

c		u		
		0	1	2
v	0	$\pi R^2/A$	$\pi R^2\overline{DBH}/A$	$\pi R^2\overline{DBH^2}/A$
	1	$2\pi R/A$	$2\pi R\overline{DBH}/A$	$2\pi R\overline{DBH^2}/A$

Supplementary Figures

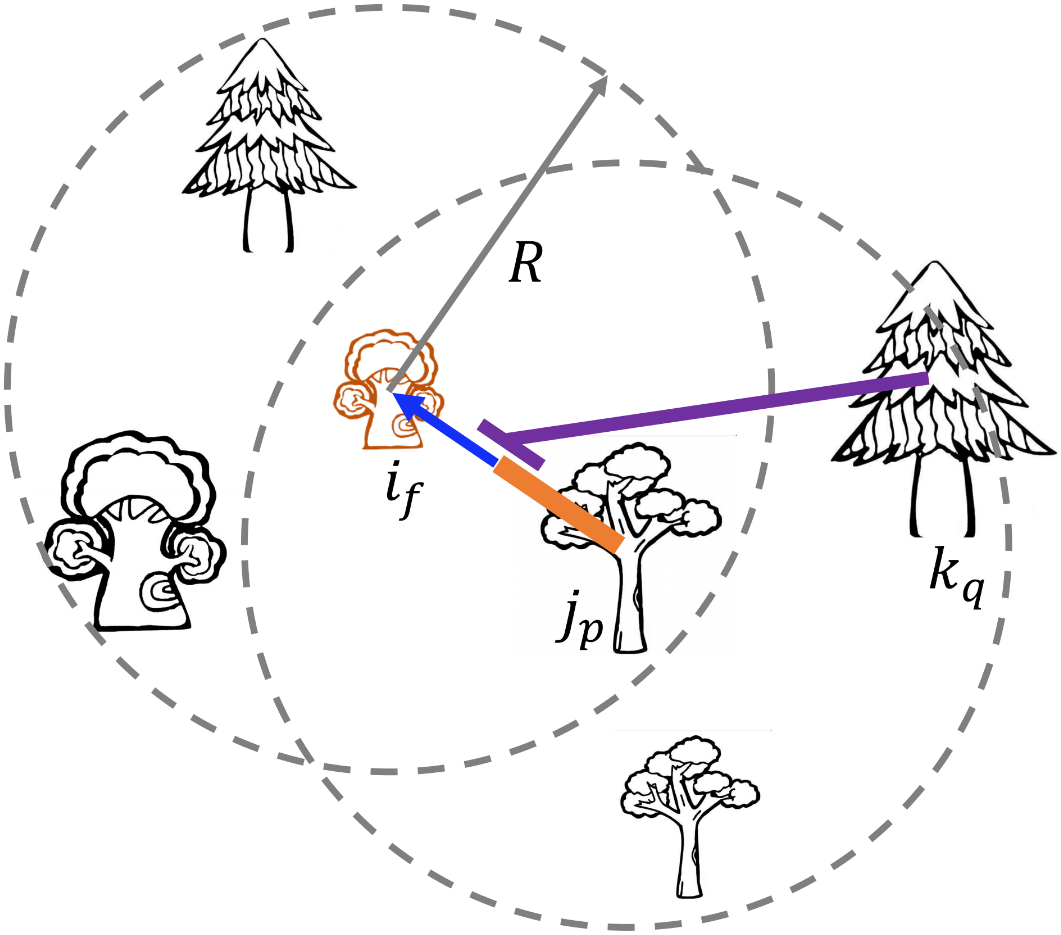

Supplementary Figure 1 | A diagram illustrating pairwise (orange arrow), higher-order (purple arrow) and total effects (blue arrow) on the focal tree $\boldsymbol{i_f}$ (tree $\boldsymbol{f}$ of species $\boldsymbol{i}$). Here, j_p (tree p of species j) is a neighbor within distance R of i_f, and k_q (tree q of species k) is a neighbor also within distance R of j_p (i.e., a neighbor's neighbor of i_f).

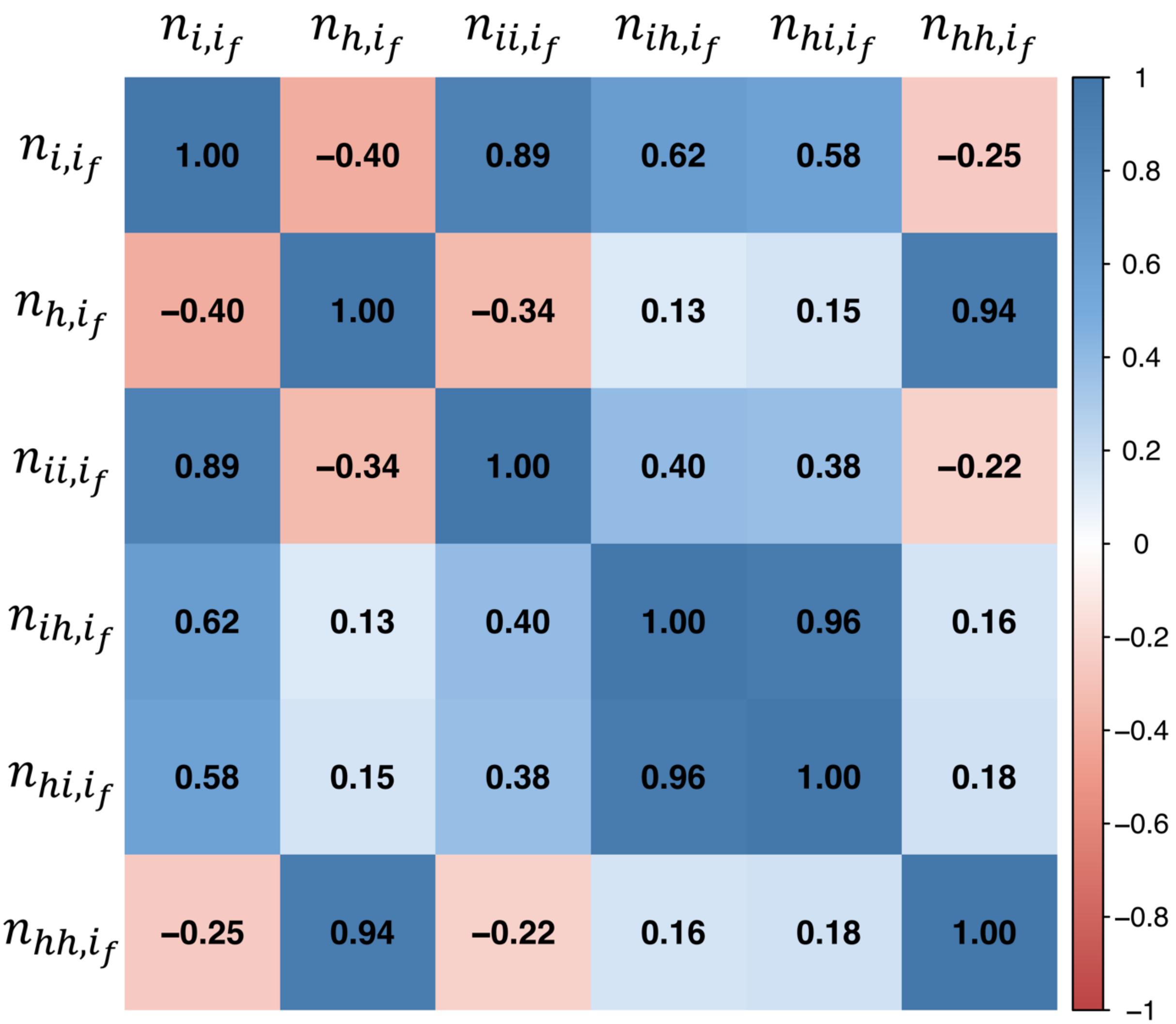

Supplementary Figure 2 | Pearson correlation coefficients between different type of neighborhood crowding indices. Here, n_{i,i_f} and n_{h,i_f} denote pairwise conspecific and heterospecific neighborhood crowding indices of focal tree i_f. The notions n_{ii,i_f}, n_{ih,i_f}, n_{hi,i_f} and n_{hh,i_f} are the four higher-order neighborhood crowding indices of focal tree i_f, corresponding to intraspecific pairwise interactions modified by conspecific neighbors ($\alpha_{ii} \leftarrow i$), intraspecific pairwise interactions modified by heterospecific neighbors ($\alpha_{ii} \leftarrow h$), interspecific pairwise interactions modified by conspecific neighbors ($\alpha_{ih} \leftarrow i$), and interspecific pairwise interactions modified by heterospecific neighbors ($\alpha_{ih} \leftarrow h$), respectively.

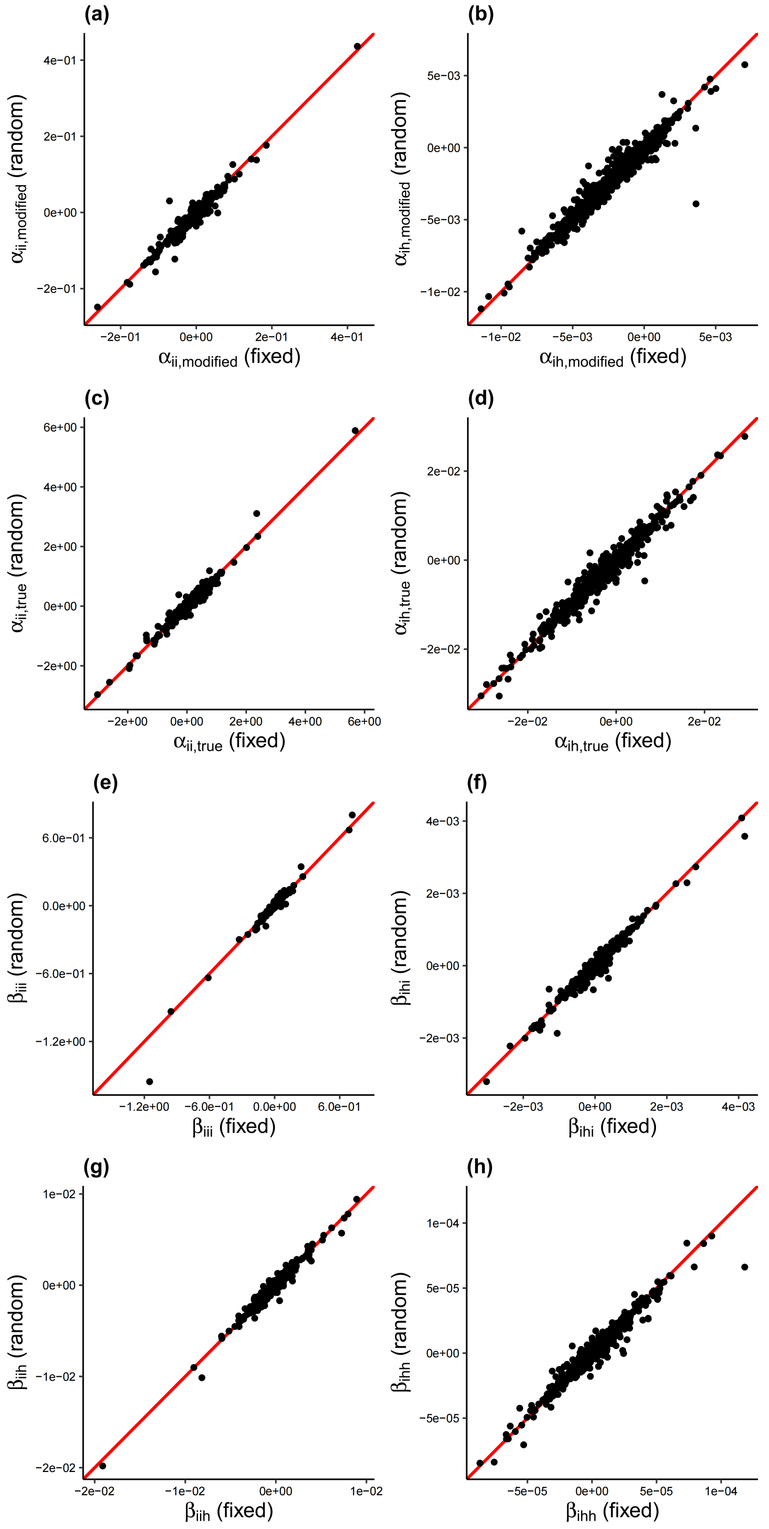

Supplementary Figure 3 | Correlations between interaction coefficients estimated from growth models with (y-axis) and without (x-axis) considering spatial autocorrelation. The red line indicates the relationship $y = x$.

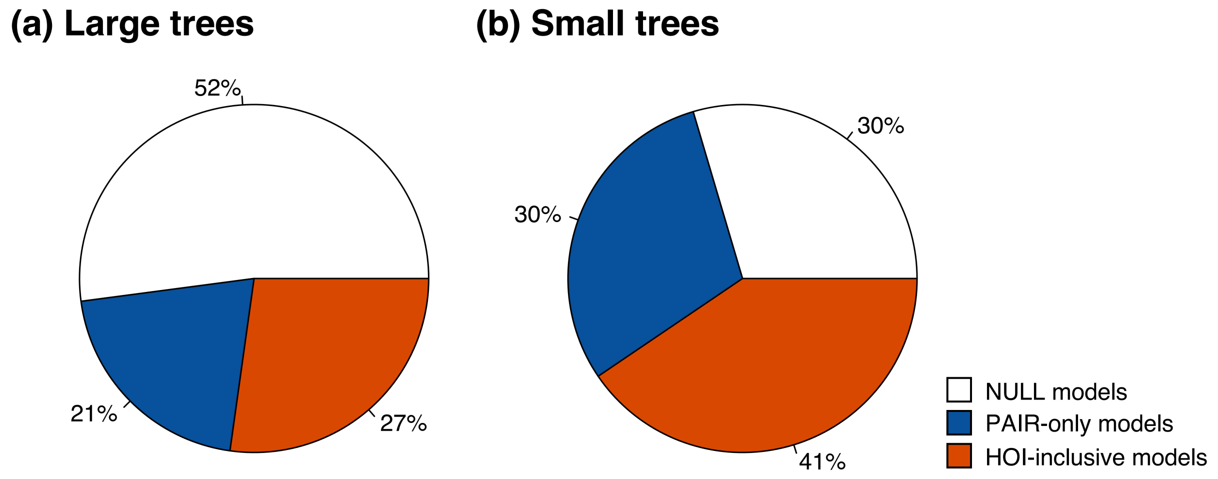

Supplementary Figure 4 | Evidence of HOIs from growth models for large ($DBH \geq 10$ cm) and small ($DBH < 10$ cm) trees. Panels (a) and (b) show the percentage of 432 and 1,362 tree species–plot combinations, respectively, across the 32 plots for which each of the three classes of growth models are best supported (AIC at least 2 units lower than that of the two alternative models). The white, blue and orange regions indicate the percentage of species supporting the NULL models (models without including biotic interactions), PAIR-only models (models includes only pairwise interactions) and the HOI-inclusive models (models include both pairwise interactions and HOIs), respectively.

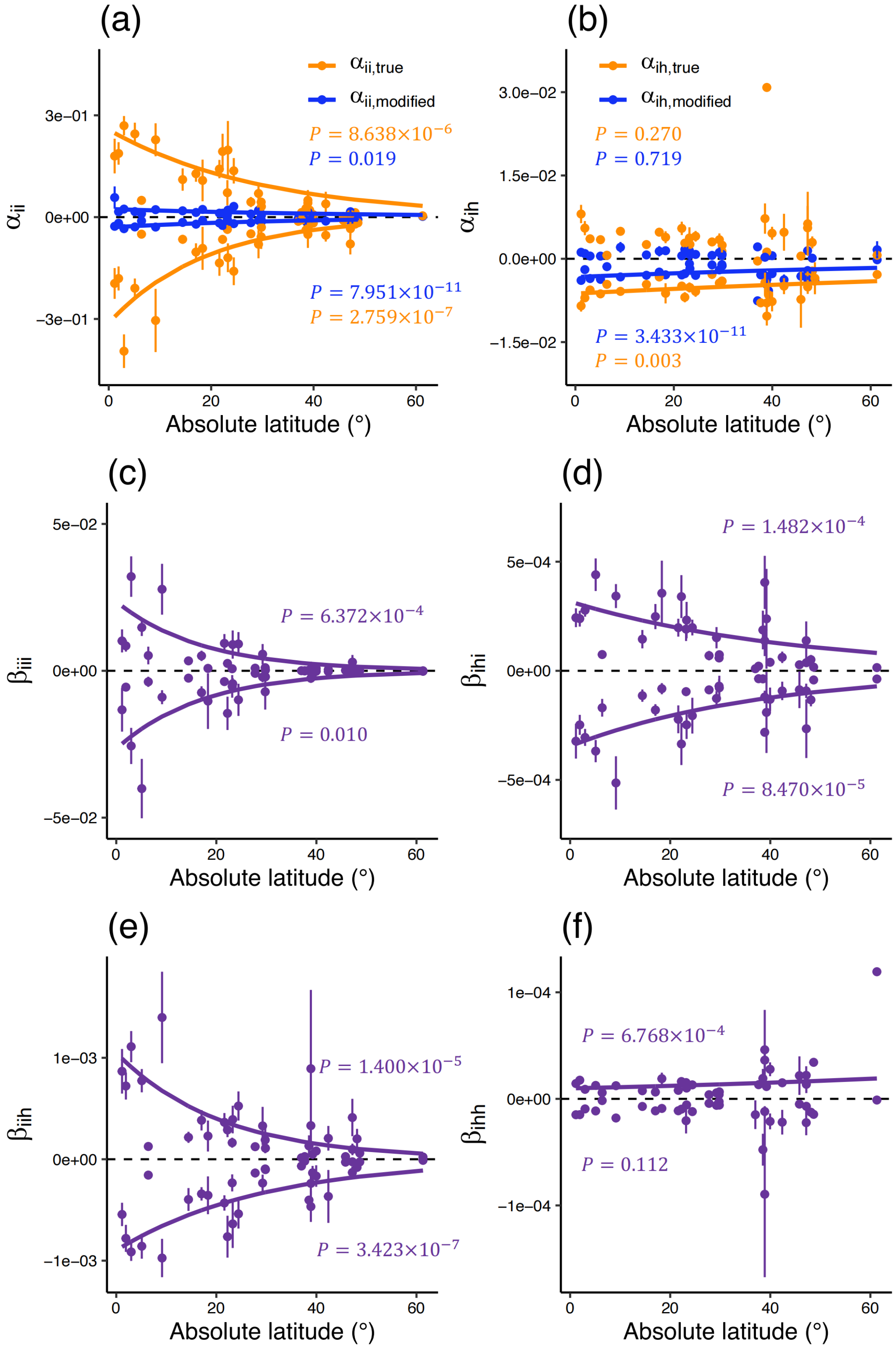
(a)
$\alpha_{ii,true}$
$\alpha_{ii,modified}$
$P = 8.638\times10^{-6}$
$P = 0.019$
$P = 7.951\times10^{-11}$
$P = 2.759\times10^{-7}$
α_{ii}
3e−01
0e+00
−3e−01
0
20
40
60
Absolute latitude (°)
(b)
$\alpha_{ih,true}$
$\alpha_{ih,modified}$
$P = 0.270$
$P = 0.719$
$P = 3.433\times10^{-11}$
$P = 0.003$
α_{ih}
3.0e−02
1.5e−02
0.0e+00
−1.5e−02
0
20
40
60
Absolute latitude (°)
(c)
$P = 6.372\times10^{-4}$
$P = 0.010$
β_{iii}
5e−02
0e+00
−5e−02
0
20
40
60
Absolute latitude (°)
(d)
$P = 1.482\times10^{-4}$
$P = 8.470\times10^{-5}$
β_{ihi}
5e−04
0e+00
−5e−04
0
20
40
60
Absolute latitude (°)
(e)
$P = 1.400\times10^{-5}$
$P = 3.423\times10^{-7}$
β_{iih}
1e−03
0e+00
−1e−03
0
20
40
60
Absolute latitude (°)
(f)
$P = 6.768\times10^{-4}$
$P = 0.112$
β_{ihh}
1e−04
0e+00
−1e−04
0
20
40
60
Absolute latitude (°)

Supplementary Figure 5 | Latitudinal changes in pairwise and higher-order interactions from growth models for small trees ($DBH < 10$ cm). Panels (a) and (b) display the latitudinal changes in intraspecific (α_{ii}) and interspecific (α_{ih}) pairwise interactions, respectively. The pairwise interactions estimated from PAIR-only models and HOI-inclusive models, denoted as $\alpha_{modified}$ and α_{true}, are distinguished by blue and orange points (lines). Panels (c) and (e) show the latitudinal changes in higher-order interaction coefficients β_{iii} and β_{iih}, which represent the modifications of intraspecific pairwise interactions by conspecific neighbors ($\alpha_{ii} \leftarrow i$) and by heterospecific neighbors ($\alpha_{ii} \leftarrow h$), respectively. Panels (d) and (f) show the latitudinal changes in higher-order interaction coefficients β_{iii} and β_{iih}, corresponding to the modifications of interspecific pairwise interactions by conspecific neighbors ($\alpha_{ih} \leftarrow i$) and heterospecific neighbors ($\alpha_{ih} \leftarrow h$), respectively. Species-level pairwise and higher-order interactions are related to absolute latitude separately for competitive ($\alpha < 0, \beta < 0$) and facilitative interactions ($\alpha > 0, \beta > 0$) using exponential regressions. Significance of the regression coefficients is assessed using two-sided *t*-test; exact *P* values are shown in the figure. The regression lines are shown only when the interaction strength significantly changes with latitude (p < 0.05). For clarity, we display plot-level mean values +/- SEM rather than species-level estimates.

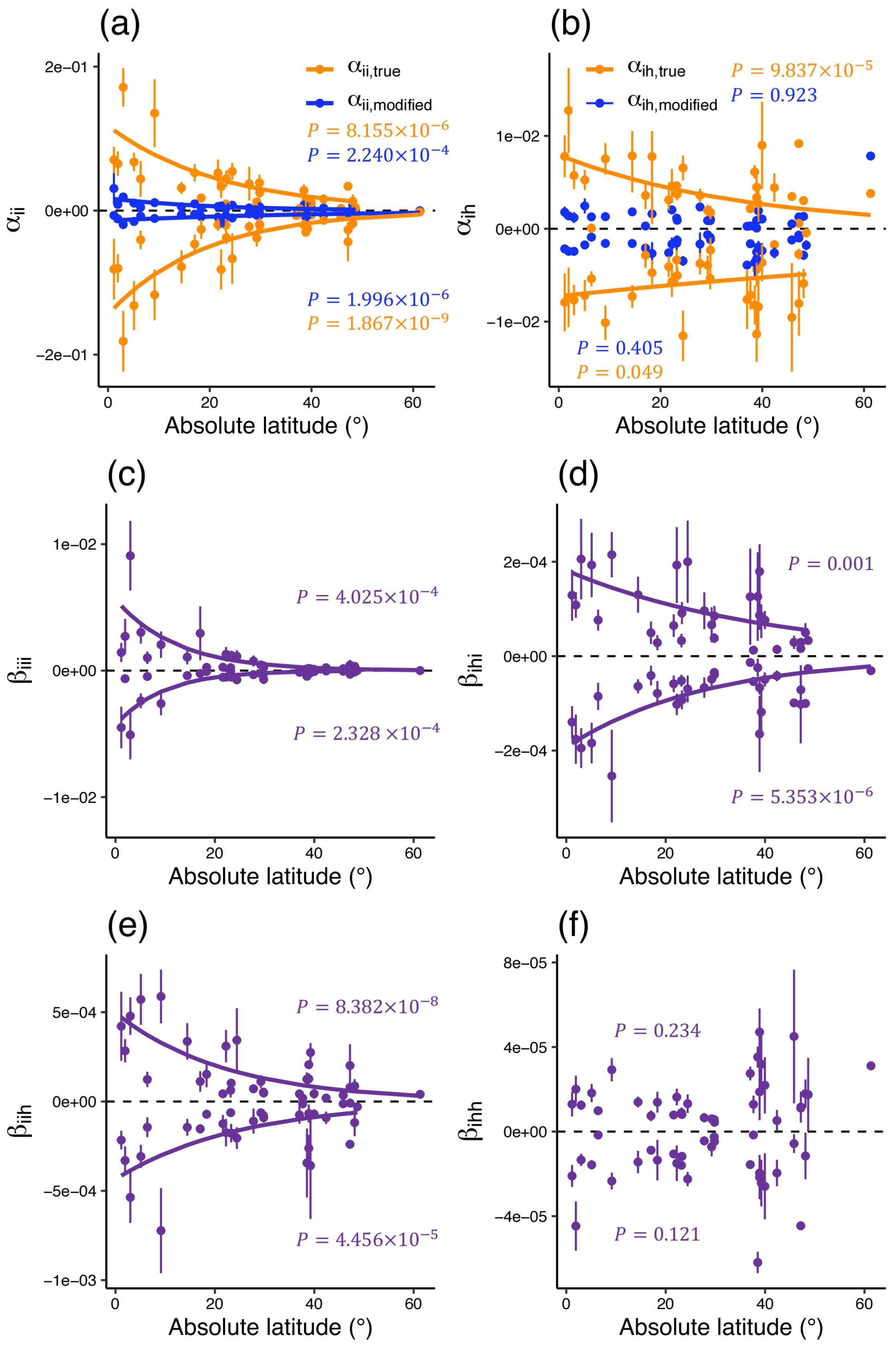
(a)
2e−01
0e+00
−2e−01
αii
αii,true
αii,modified
P = 8.155×10−6
P = 2.240×10−4
P = 1.996×10−6
P = 1.867×10−9
0
20
40
60
Absolute latitude (°)
(b)
1e−02
0e+00
−1e−02
αih
αih,true
αih,modified
P = 9.837×10−5
P = 0.923
P = 0.405
P = 0.049
Absolute latitude (°)
(c)
1e−02
0e+00
−1e−02
βiii
P = 4.025×10−4
P = 2.328 ×10−4
Absolute latitude (°)
(d)
2e−04
0e+00
−2e−04
βihi
P = 0.001
P = 5.353×10−6
Absolute latitude (°)
(e)
5e−04
0e+00
−5e−04
−1e−03
βiih
P = 8.382×10−8
P = 4.456×10−5
Absolute latitude (°)
(f)
8e−05
4e−05
0e+00
−4e−05
βihh
P = 0.234
P = 0.121
Absolute latitude (°)

Supplementary Figure 6 | Latitudinal changes in pairwise and higher-order interactions (HOIs) from growth models for large trees ($DBH \geq 10$ cm). Panels (a) and (b) display the latitudinal changes in intraspecific (α_{ii}) and interspecific (α_{ih}) pairwise interactions, respectively. The pairwise interactions estimated from PAIR-only models and HOI-inclusive models, denoted as $\alpha_{modified}$ and α_{true}, are distinguished by blue and orange points (lines). Panels (c) and (e) show the latitudinal changes in higher-order interaction coefficients β_{iii} and β_{iih}, which represent the modifications of intraspecific pairwise interactions by conspecific neighbors ($\alpha_{ii} \leftarrow i$) and by heterospecific neighbors ($\alpha_{ii} \leftarrow h$), respectively. Panels (d) and (f) show the latitudinal changes in higher-order interaction coefficients β_{iii} and β_{iih}, corresponding to the modifications of interspecific pairwise interactions by conspecific neighbors ($\alpha_{ih} \leftarrow i$) and heterospecific neighbors ($\alpha_{ih} \leftarrow h$), respectively. Species-level pairwise and higher-order interactions are related to absolute latitude separately for competitive ($\alpha < 0, \beta < 0$) and facilitative interactions ($\alpha > 0, \beta > 0$) using exponential regressions. Significance of the regression coefficients is assessed using two-sided *t*-test; exact *P* values are shown in the figure. The regression lines are shown only when the interaction strength significantly changes with latitude (p < 0.05). For clarity, we display plot-level mean values +/- SEM rather than species-level estimates.

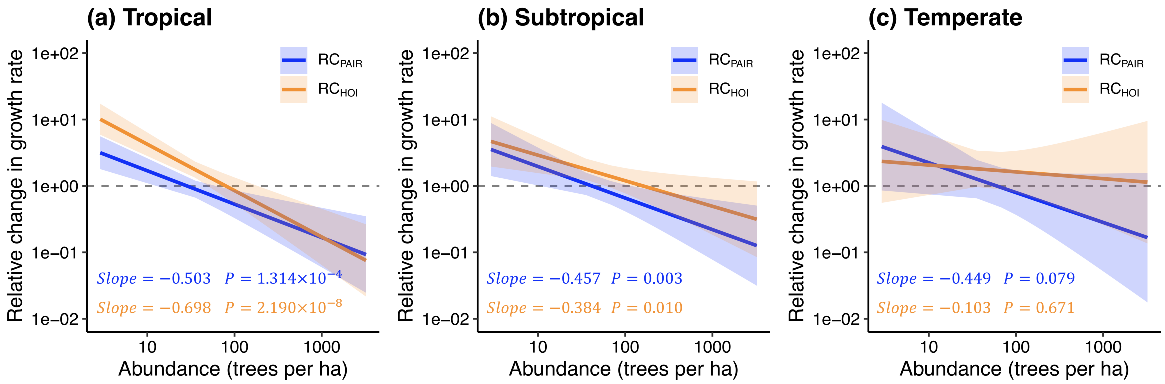

Supplementary Figure 7 | Predicted relationships between relative change in growth rate and species abundance across three latitudinal geographic zones for small trees ($DBH <$ 10 cm). RC_{PAIR} and RC_{HOI} that represent the relative changes in growth rate caused by cumulative effects of pairwise interactions and HOIs, are distinguished by blue and orange lines. Predictions are shown for three geographic zones: tropical (0°–23.5°, a), subtropical (23.5°–35°, b), and temperate (35°–66.5°, c). Predictions are generated from the linear models summarized in Supplementary Table 8, using the middle latitude of each zone (i.e., 11.75° for the tropical zone, 29.25° for the subtropical zone and 45° for the temperate zone). Solid lines show model predictions and shaded areas represent 95% confidence intervals.

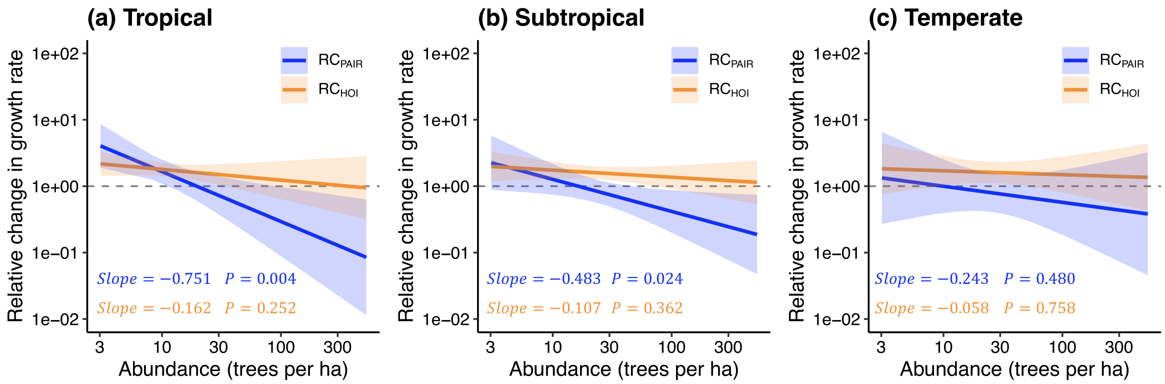

Supplementary Figure 8 | Predicted relationships between relative change in growth rate and species abundance across three latitudinal geographic zones for large trees ($DBH \geq$ 10 cm). RC_{PAIR} and RC_{HOI} that represent the relative changes in growth rate caused by cumulative effects of pairwise interactions and HOIs, are distinguished by blue and orange lines. Predictions are shown for three geographic zones: tropical (0°–23.5°, a), subtropical (23.5°–35°, b), and temperate (35°–66.5°, c). Predictions are generated from the linear models summarized in Supplementary Table 9, using the middle latitude of each zone (i.e., 11.75° for the tropical zone, 29.25° for the subtropical zone and 45° for the temperate zone). Solid lines show model predictions and shaded areas represent 95% confidence intervals.

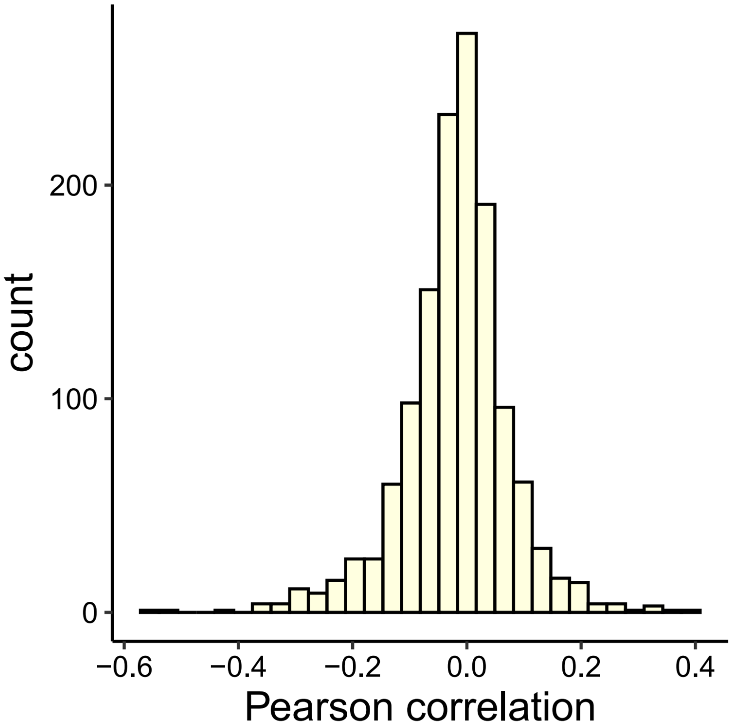

Supplementary Figure 9 | Distribution of Pearson correlations between density-independent (a) and density-dependent (x) effects survival probability for 1340 species–plot combinations.

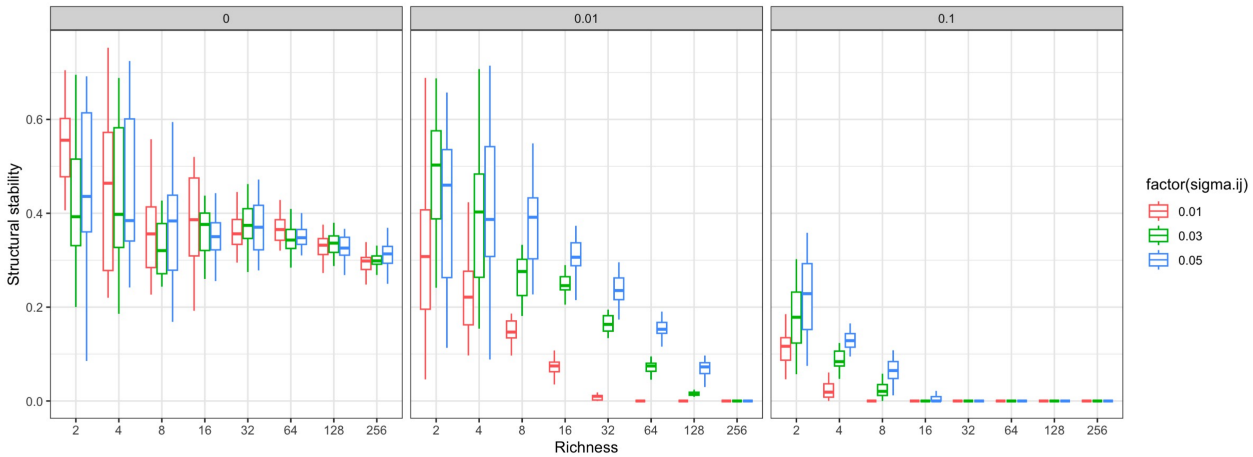

Supplementary Figure 10 | Theoretical relationship between richness and structural stability for random interactions. The relationship between richness and structural stability for random interactions. The interaction matrix $[\alpha_{ij}]_{n\times n}$ was randomly sampled from a normal distribution with mean of mu_{ij} and standard deviation of $sigma_{ij}$ for a given richness (n). Different panels indicate different mean interaction strengths ($mu_{ij} = 0, 0.1, 0.01$). Different colors indicate different variations of interaction strength ($sigma_{ij} = 0.01, 0.03, 0.05$). Structural stability declines with species richness and reaches zero at high richness except for when $mu_{ij} = 0$.

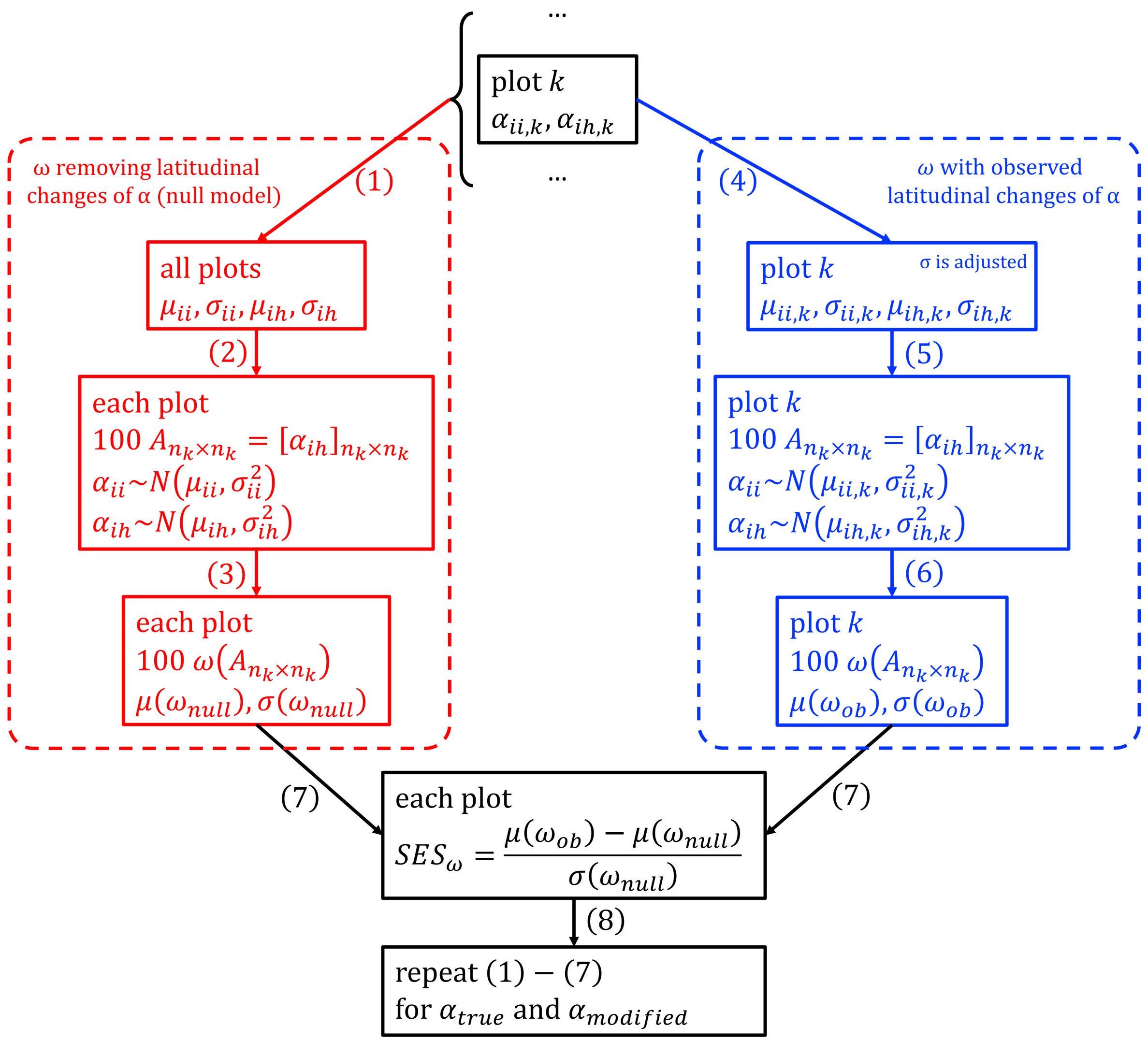

Supplementary Figure 11 | The diagram of how we constructed null models for the structural stability analysis for each forest plot. The red parts are steps for calculating structural stability (ω_{null}) of null models where species interactions are sampled from global distributions of intraspecific and interspecific interactions determined by their means (μ_{ii}, μ_{ih}) and standard deviations (σ_{ii}, σ_{ih}). The blue parts are steps for calculating observed structural stability (ω_{ob}) where species interactions are sampled from plot specific distributions of intraspecific and interspecific interactions determined by their means ($\mu_{ii,k}, \mu_{ih,k}$) and standard deviations ($\sigma_{ii,k}, \sigma_{ih,k}$).

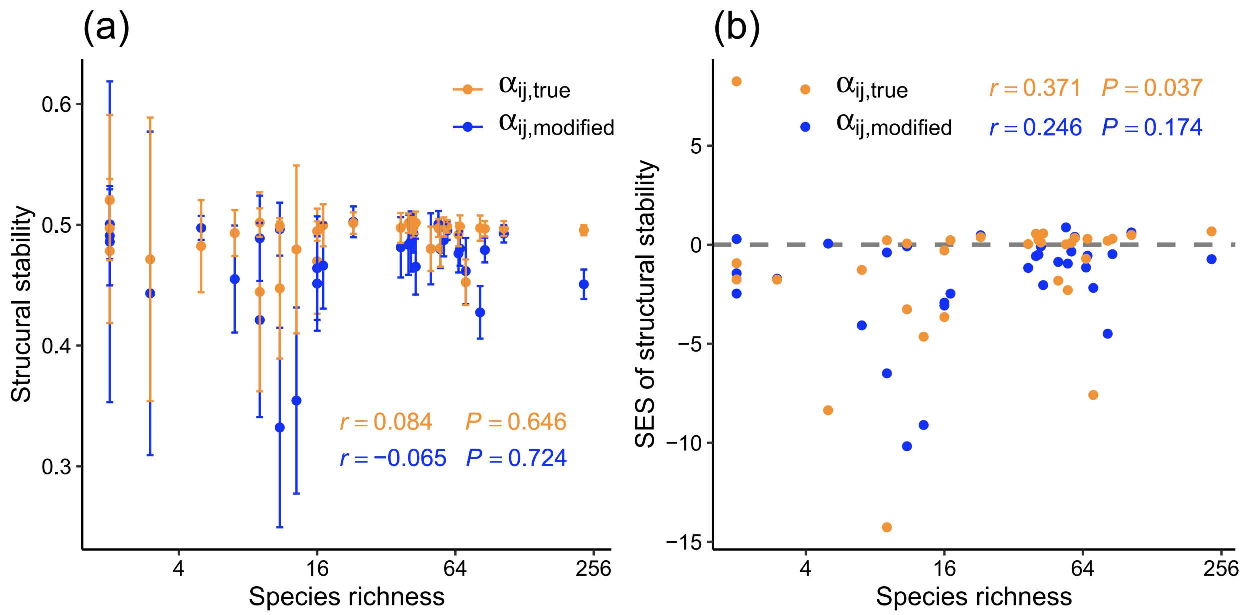

Supplementary Figure 12 | Variation of observed structural stability (a) and standard effect sizes (b) with species richness. Structural stability and its standard effect size based on $\alpha_{ij,true}$ and $\alpha_{ij,modified}$, that are estimated from HOI-inclusive and PAIR-only models, are distinguished by orange and blue colors. Spearman rank correlation and *P* values are provided in the figure.
