# Supplementary material for: Higher-order interactions enhance the latitudinal tree diversity gradient
Source: Nature. 2026 Apr 29;653(8114):433–8. doi: 10.1038/s41586-026-10434-6 (PMC13171435; doi:10.1038/s41586-026-10434-6)
Supplement: Supplementary file 2 — Reporting Summary [file 41586_2026_10434_MOESM2_ESM.pdf]

nature portfolio

Corresponding author(s): Chengjin Chu

Last updated by author(s): Mar 17, 2026

Reporting Summary

Nature Portfolio wishes to improve the reproducibility of the work that we publish. This form provides structure for consistency and transparency in reporting. For further information on Nature Portfolio policies, see our Editorial Policies and the Editorial Policy Checklist.

Statistics

For all statistical analyses, confirm that the following items are present in the figure legend, table legend, main text, or Methods section.

n/a	Confirmed	
☐	☒	The exact sample size (n) for each experimental group/condition, given as a discrete number and unit of measurement
☐	☒	A statement on whether measurements were taken from distinct samples or whether the same sample was measured repeatedly
☐	☒	The statistical test(s) used AND whether they are one- or two-sided<br>*Only common tests should be described solely by name; describe more complex techniques in the Methods section.*
☐	☒	A description of all covariates tested
☐	☒	A description of any assumptions or corrections, such as tests of normality and adjustment for multiple comparisons
☐	☒	A full description of the statistical parameters including central tendency (e.g. means) or other basic estimates (e.g. regression coefficient) AND variation (e.g. standard deviation) or associated estimates of uncertainty (e.g. confidence intervals)
☐	☒	For null hypothesis testing, the test statistic (e.g. F, t, r) with confidence intervals, effect sizes, degrees of freedom and P value noted<br>*Give P values as exact values whenever suitable.*
☒	☐	For Bayesian analysis, information on the choice of priors and Markov chain Monte Carlo settings
☒	☐	For hierarchical and complex designs, identification of the appropriate level for tests and full reporting of outcomes
☐	☒	Estimates of effect sizes (e.g. Cohen's d, Pearson's r), indicating how they were calculated

Our web collection on statistics for biologists contains articles on many of the points above.

Software and code

Policy information about availability of computer code

Data collection	No software was used for data collection.
Data analysis	Data analyses were conducted in R version 4.4.1 using several packages, including DHARMa (v0.4.7), minpack.lm (v1.2-4) and ggplot2(v4.0.0). Additional packages used in the analyses are documented in the code. The custom code used in this study is available at Figshare: https://doi.org/10.6084/m9.figshare.28426862.

For manuscripts utilizing custom algorithms or software that are central to the research but not yet described in published literature, software must be made available to editors and reviewers. We strongly encourage code deposition in a community repository (e.g. GitHub). See the Nature Portfolio guidelines for submitting code & software for further information.

Data

Policy information about availability of data

All manuscripts must include a data availability statement. This statement should provide the following information, where applicable:

- Accession codes, unique identifiers, or web links for publicly available datasets
- A description of any restrictions on data availability
- For clinical datasets or third party data, please ensure that the statement adheres to our policy

The raw census data that support this study are available upon request and with permission of the principal investigators of the ForestGEO and CForBio networks

(names and contact information of the PIs are provided in Supplementary Table 1). For some plots, the data are publicly available at https://forestgeo.si.edu/explore-data. The processed datasets supporting the findings of this study are publicly available at Figshare: https://doi.org/10.6084/m9.figshare.28426862.

Research involving human participants, their data, or biological material

Policy information about studies with human participants or human data. See also policy information about sex, gender (identity/presentation), and sexual orientation and race, ethnicity and racism.

Reporting on sex and gender	Not applicable.
Reporting on race, ethnicity, or other socially relevant groupings	Not applicable.
Population characteristics	Not applicable.
Recruitment	Not applicable.
Ethics oversight	Not applicable.

Note that full information on the approval of the study protocol must also be provided in the manuscript.

Field-specific reporting

Please select the one below that is the best fit for your research. If you are not sure, read the appropriate sections before making your selection.

☐ Life sciences ☐ Behavioural & social sciences ☒ Ecological, evolutionary & environmental sciences

For a reference copy of the document with all sections, see nature.com/documents/nr-reporting-summary-flat.pdf

Ecological, evolutionary & environmental sciences study design

All studies must disclose on these points even when the disclosure is negative.

Study description	In this study, we assembled census data from 32 large permanent forest plots spanning tropical to boreal forests to address three key questions: (Q1) Are HOIs prevalent among trees across forest plots? (Q2) How do pairwise interactions and HOIs vary with latitude? (Q3) How do latitudinal changes in HOIs contribute to the latitudinal tree diversity gradient? To answer these questions, we estimated both pairwise interactions and HOIs from demographic growth (for 1,543 tree species–plot combinations) and survival models (for 1,340 tree species–plot combinations), respectively. With these data, we built three types of growth and survival models: (i) NULL models with no biotic interactions, (ii) PAIR-only models including only pairwise interactions, and (iii) HOI-inclusive models including both pairwise interactions and HOIs. We then compared AIC of the three types of models (Q1), tested whether the estimated pairwise interactions and HOIs declined with latitudes (Q2), and evaluated how the cumulative effects of pairwise interactions and HOIs on growth and survival changed with species abundance and latitude (Q3).
Research sample	The data used in this study were collected at 32 large permanent forest dynamic plots worldwide from the Forest Global Earth Observatory (ForestGEO, http://www. forestgeo.si.edu) and Chinese Forest Biodiversity Monitoring Network (CForBio, http://www.cfbiodiv.org).
Sampling strategy	Most plots had only been censused twice. For a few plots with three or more censuses, we selected two consecutive censuses between 1998 and 2022 for analysis. Overall, we compiled data for over three million trees of 5,000 species across 32 plots.
Data collection	All plots were established and censused multiple times following a standardized protocol. In each census, all free-standing woody stems with a diameter at breast height (DBH) larger than 1 cm were tagged (unique ID), mapped (coordinates), identified (species identity), measured (DBH), and recorded (alive, dead or recruit). The census was repeated every 5 years to monitor forest dynamics (e.g., survival, growth, and recruitment).
Timing and spatial scale	The 32 forest plots span tropical to boreal terrestrial biomes with latitude ranging from 1.92° S to 61.30° N. These plots vary in size between 9 and 50 ha (24.5 ha on average). At all plots included in this study, two ore more censuses have been carried out with remeasurement intervals of approximately five years. Most plots had only been censused twice. For a few plots with three or more censuses, we selected two consecutive censuses between 1998 and 2022 for analysis.
Data exclusions	Observations of trees were excluded when information on coordinates, species, or status was missing. We fit demographic growth and survival models for each species with more than 100 trees (and additionally with at least 20 alive and dead observations for the survival model) to ensure model performance and robustness.
Reproducibility	This study is based on observational data, and no experiments are conducted.
Randomization	This study is based on observational data. Therefore, randomization into groups does not apply.

Blinding | Blinding is not relevant for this study because the data were not specifically collected to assess direct and higher-order interactions.

Did the study involve field work? ☐ Yes ☒ No

Reporting for specific materials, systems and methods

We require information from authors about some types of materials, experimental systems and methods used in many studies. Here, indicate whether each material, system or method listed is relevant to your study. If you are not sure if a list item applies to your research, read the appropriate section before selecting a response.

Materials & experimental systems

n/a	Involved in the study
☒	☐ Antibodies
☒	☐ Eukaryotic cell lines
☒	☐ Palaeontology and archaeology
☒	☐ Animals and other organisms
☒	☐ Clinical data
☒	☐ Dual use research of concern
☒	☐ Plants

Methods

n/a	Involved in the study
☒	☐ ChIP-seq
☒	☐ Flow cytometry
☒	☐ MRI-based neuroimaging

Plants

Seed stocks | Not applicable.

Novel plant genotypes | Not applicable.

Authentication | Not applicable.
